# Sequence motifs associated with hepatotoxicity of locked nucleic acid—modified antisense oligonucleotides

**DOI:** 10.1093/nar/gku142

**Published:** 2014-02-18

**Authors:** Andrew D. Burdick, Simone Sciabola, Srinivasa R. Mantena, Brett D. Hollingshead, Robert Stanton, James A. Warneke, Ming Zeng, Elena Martsen, Alexander Medvedev, Sergei S. Makarov, Lori A. Reed, John W. Davis, Laurence O. Whiteley

**Affiliations:** ^1^Drug Safety Research and Development, Worldwide Research and Development, Pfizer Inc., Cambridge, MA 02140, USA, ^2^Oligonucleotide Therapeutic Unit, Worldwide Research and Development, Pfizer Inc., Cambridge, MA 02139, USA and ^3^Attagene Inc., Morrisville, NC 27560, USA

## Abstract

Fully phosphorothioate antisense oligonucleotides (ASOs) with locked nucleic acids (LNAs) improve target affinity, RNase H activation and stability. LNA modified ASOs can cause hepatotoxicity, and this risk is currently not fully understood. *In vitro* cytotoxicity screens have not been reliable predictors of hepatic toxicity in non-clinical testing; however, mice are considered to be a sensitive test species. To better understand the relationship between nucleotide sequence and hepatotoxicity, a structure–toxicity analysis was performed using results from 2 week repeated-dose-tolerability studies in mice administered LNA-modified ASOs. ASOs targeting human Apolipoprotien C3 (*Apoc3*), CREB (cAMP Response Element Binding Protein) Regulated Transcription Coactivator 2 (*Crtc2*) or Glucocorticoid Receptor (*GR*, *NR3C1*) were classified based upon the presence or absence of hepatotoxicity in mice. From these data, a random-decision forest-classification model generated from nucleotide sequence descriptors identified two trinucleotide motifs (TCC and TGC) that were present only in hepatotoxic sequences. We found that motif containing sequences were more likely to bind to hepatocellular proteins *in vitro* and increased P53 and NRF2 stress pathway activity *in vivo*. These results suggest *in silico* approaches can be utilized to establish structure–toxicity relationships of LNA-modified ASOs and decrease the likelihood of hepatotoxicity in preclinical testing.

## INTRODUCTION

The development of RNaseH dependent antisense therapeutics was initially hindered by the stability and potency of DNA oligonucleotides (ONs). Advances in oligonucleotide chemistry have significantly improved target specificity, pharmacokinetics (PK), pharmacodynamics (PD), RNA-silencing efficiency and nuclease degradation properties. Many variations of base, sugar and backbone modifications have evolved and several have advanced to the clinic including the phosphorothioate (PS) backbone modification (1), 2′-*O*-methyl (2′-OMe) (2) and 2′-*O*-methoxyethyl (2′-MOE) substitutions (3), and the 2′-O, 4′-C-methylene linked bicyclic ribofuranosyl modification (locked nucleic acid, LNA) (4–9). Of importance to recent RNaseH mediated antisense oligonucleotides (ASOs) was the adoption of ‘gapmer’ modification patterns which use a central string of DNA nucleotides flanked by higher affinity chemically modified nucleotides such as LNA or MOE (10). The most clinically advanced antisense compounds include LNA gapmers from Enzon (e.g. EZN-2968 an inhibitor of *HIF1α* for oncology) (11), MOE gapmers from ISIS (e.g. mipomersen targeting *ApoB* as a lipid-lowering agent) (12,13) and LNA mixmers from Santaris (e.g. miraversen a blocker of miR-122 as a treatment for HCV) (14,15).

With the LNA modifications, a methylene bridge locks the ribose in the 3′-endo (N-type) confirmation and substantially improves duplex stability to complimentary DNA or RNA by enhancing base stacking properties (16). Although LNA modifications offer advantages in target affinity and stability, these modifications were reported to cause increased hepatotoxicity in mice compared to sequences with 2′-MOE modifications. Swayze *et al.* (17) reported previously that mice treated with LNA-modified ONs had significantly increased plasma aminotransferase (ALT) and aspartate aminotransferase (AST) levels as well as histopathological evidence of liver necrosis and activation of apoptosis. This article linked hepatotoxicity to the LNA modification, and proposed that toxicity was not related to target knockdown or off-target gene silencing. More recently, Hagedorn *et al.*, unequivolently demonstrated that there is no relationship between target transcript levels and the hepatotoxicity of LNAs (18). Furthermore, while the LNA modification is strongly associated with increased toxicity, it cannot be said that all LNA-modified sequences are universally hepatoxic since several of these therapeutics have successfully advanced through preclinical safety studies (14,15).

Our experience with LNA-modified ASOs is similar to these previous reports regarding the propensity for LNA modifications to cause liver toxicity in mice. For example, we found that approximately half of all LNA-modified sequences designed to target either human *Apoc3*, *Crtc2* or *GR* were hepatotoxic when screened for tolerability in mice. The toxicity we observed was dependent on the ASO design because subtle changes in the sequence, such as a shift up or down the target region, and/or a change to the gapmer configuration, could eliminate the toxicity without affecting target potency (10). These clues pointing towards sequence-dependent toxicity suggested that structure activity approaches could be used to predict the toxicity from the sequence or the chemical modification pattern.

In support of this hypothesis, Hagedorn *et al.* were first to report that structural descriptors could, in fact, be used to predict toxicity of LNA-modified ASOs (18). Using sequences consisting of a central DNA region of at least seven nucleotides flanked by either two or three LNAs, sequence descriptors consisting of dinucleotide repeats were used to train a random forests classification algorithm. Their model identified as many as 17 dinucleotide motifs when present alone, or in combination, could distinguish between sequences with high or low hepatotoxicity potential (18).

In this article, we applied similar machine learning techniques to 3LNA-8DNA-3LNA (3-8-3) gapmer designs to test whether sequence descriptors could be used to predict toxicity outcome. We further expand on the work of Hagedorn *et al.* by reporting several important additional observations. Here, we report a strong relationship between hepatotoxicity in mice to two specific trinucleotide sequence motifs (TGC and/or TCC) in oligonucleotides with the 3-8-3 design. In addition, sequences that carry these motifs have a higher propensity to bind to hepatocellular proteins *in vitro*, suggesting a potential toxicity mechanism. Finally, we found that these motif-containing sequences increased P53- and Nrf2-dependent transcriptional pathways in the liver, indicating a linkage between stress pathway activation and hepatotoxicity. Our results demonstrate that structure toxicity relationships can be carried out with LNA-modified ONs, allowing for potentially toxic sequence motifs to be eliminated at the design stage, thereby reducing development cost and the need for extensive tolerability screening in mice.

## MATERIALS AND METHODS

### Design and synthesis of LNA-modified antisense oligonucleotides

An in-house design workflow was applied for the selection of all antisense ONs. The selection algorithm used the longest human splice variant for each gene enumerated as 14-mer antisense. Final selection of ONs for potency and pharmacology studies takes into account multiple factors including: homology with other human splice variants and orthologs (mouse, rat and macaque); known human SNPs; sequence motifs associated with non-specific binding; and low-complexity regions in the mRNA. The chosen sequences were chemically modified to have a phosphorothioated backbone and the 3-8-3 LNA gapmer design. Additionally, any CpG motifs were methylated to reduce the potential for any immune stimulatory response. All oligonucleotides were synthesized as previously reported (10).

### Toxicity studies in CD-1 mice

The Pfizer Institutional Animal Care and Use Committee (IACUC) reviewed and approved the animal use in these studies. The animal care and use program is fully accredited by the Association for Assessment and Accreditation of Laboratory Animal Care, International. Studies of 2 weeks in duration were used to evaluate the potential hepatotoxicity of ASOs. Male CD-1 mice aged 5–7-weeks old were obtained from Charles River Laboratories. ASOs or 0.9% saline alone were administered subcutaneously in the scapular region of the mouse at a dose volume of 10 ml/kg on study days 1, 4, 8 and 11. The final dosage of ASOs was 25 mg/kg based on the most recent body weight. On study-day 15, animals were anesthetized by inhalation of 2–4% isoflurane and blood was collected via the vena cava. Blood was processed to serum and evaluated for the following parameters: ALT, alkaline phosphatase (ALP), AST, total bilirubin, glutamate dehydrogenase (GLDH) and troponin. Livers were collected and weighed and subsequently embedded in paraffin and stained with hematoxylin and eosin using standard procedures. Microscopic evaluation of tissues was conducted by a board certified veterinary pathologist. Sequences were classified as having lesions (toxic) or no lesions (non-toxic) based on presence or absence of one or more of the following: single-cell necrosis, mitoses, karyomegaly/cytomegaly, hepatocellular hypertrophy and hepatocellular necrosis. Lesions were graded as minimal, mild, moderate or severe. The most severe grade (among four or five animals in each group) for each of these lesions was used in the final classification of lesions for each of the sequences and is presented in Supplementary Material.

### Classification model for identifying structure–toxicity relationships

The dataset used in this analysis consisted of 20 non-hepatotoxic sequences and 51 sequences annotated with liver lesions of different severity. All the sequences were 3-8-3 LNA gapmers, with a central core of eight DNA nucleotides and three LNA-modified nucleosides at each end. As the design was consistent for all the 71 sequences, the effect of the modification was not included in the sequence descriptors. The characterization of the antisense sequences in terms of global contents of specific short-nucleotide motifs was done by using a numerical vector of count of occurrences of each nucleotide motif of length 2–5. Given that there are respectively 16, 64, 256, 1024 potential nucleotide motifs with length 2, 3, 4 and 5, their occurrences were encoded in an ordered vector of length 1360 (16 + 64 + 256 + 1024). To check the significance of the association of each sequence motif with the *in vivo* toxicology outcome (toxic or non-toxic), a *χ*^2^ test was used. Only the subset of variables found to be significant in the *χ*^2^ test were used to build a Random Forest classification model using all the 71 antisense gapmer sequences. We used the randomForest interface in R to the Fortran programs by Breiman and Cutler (19,20). Data was loaded in R and the *n*_tree_ parameter was set to 1000. For the *m*_try_ parameter we used the default value which samples p/3 randomly selected variables as candidates at each split.

### [γ ^32^P]ATP labeling of ASOs and protein binding

Freshly isolated untreated CD-1 mouse liver samples were homogenized in cold Dulbecco’s PBS (DPBS) containing protease inhibitor cocktail (Roche) with a glass/Teflon homogenizer. Homogenates were centrifuged at 4°C for 20 min at 21 000× *g* to remove insoluble cellular debris. Next, the protein-containing supernatant was transferred to fresh tubes, and protein concentration was determined by the BCA assay (Pierce). Protein concentrations were adjusted to 4 mg/ml with DPBS and glycerol at 1:0.6 (lysate:glycerol) ratio. Samples were aliquoted into single dispense tubes and then stored at −80°C. LNA-modified ASOs were 5′-end labeled with [γ ^32^P]ATP (6000 Ci/mmol, 10 mCi/mL, Perkin Elmer) and T4 polynucleotide kinase with the KinaseMax™ kit (Ambion) according the supplied protocol with one modification. Binding reactions were set up in 20 µl reactions by mixing of 30 000 cpm (∼1–5 pmol) of LNA compounds with 40 µg of prepared liver lysate. The reactions were incubated for 30 min at room temperature and subsequently resolved by 6% non-denaturing polyacrylamide gel electrophoresis. Gels were dried using a BioRad gel drier and exposed on phosphor imaging screens overnight. The phosphor screens were scanned and quantified using the Perkin Elmer Cyclone Plus® storage phosphor system and associated software.

### Multiplexed FACTORIAL™ analysis of transcription factor activity in the mouse liver

A FACTORIAL™ library (Attagene, Inc., www.attagene.com) comprising 46 transcription factor (TF)-responsive reporter plasmids was transfected in vivo into CD-1 mice by a ‘hydrodynamic’ transfection through the tail vein that predominantly targets the plasmids to liver parenchymal cells (21,22). Mice were allowed to recover from transfection for 2 weeks and then were treated with a single s.c. dose of saline or 25 mg/kg compound 1a, 1b or 3. Twenty-four hours later, the animals were euthanized and necropsied. Reporter RNAs’ concentrations were evaluated in total liver mRNA using a FACTORIAL™ detection protocol, as described (23). Changes in TF activities were calculated by normalizing reporter RNA concentrations in the livers of mice treated with ASOs to those in saline-treated mice. Blood for clinical chemistry and liver for microscopic examination was collected as described above. The remaining liver was immediately flash frozen in liquid nitrogen for gene expression analysis.

### Liver RNA isolation

Total RNA was isolated by homogenizing the entire left lateral lobe in 20 ml of Trizol® reagent (Invitrogen) using a handheld tissue homogenizer. Of the homogenized liver, 2 ml was cleared of insoluble material by centrifuging at 12 000× g for 10 min at 4°C, and the remaining sample was stored at −80°C for future use. The cleared homogenate was transferred to new tubes, and total RNA was isolated using the standard procedure for chloroform phase separation and isopropanol precipitation. RNA was quantified by UV absorbance (Nanodrop 8000, Thermo Scientific). Total RNA (500–1000 µg) was suspended in nuclease-free water and mRNA purified using the Poly(A)Purist™ MAG Kit (Ambion) according to the supplied instructions. The resulting mRNA was suspended in water and quantified by UV absorbance. The mRNA was shipped frozen at −80°C to Attagene for analyzing TF activities

## RESULTS

### Evaluation of hepatotoxicity in mice

Promising sequences that demonstrated sufficient *in vitro* human target potency were evaluated *in vivo* for 2 weeks in mice. Sequence selection and methods for determining potency have been described elsewhere(10). Mice were administered each ASO at 25 mg/kg s.c. on days 1, 4, 8 and 11, and then mice were euthanized on day 15 for clinical chemistry and histopathology. Based on internal experience as well as other published reports (17), this dose level and dosing frequency was expected to provide sufficient exposure to identify hepatotoxic sequences with a low number of false negative results. Over 80 unique 3-8-3 gapmers targeting the mRNAs for human *Apoc3*, *Crtc2* or *GR* were tested for tolerability *in vivo*. A range of observations, from no observable signs of toxicity to severe physical signs (morbidity/mortality), were noted (data not shown). Because all sequences were of the same 3-8-3 design and given at the same dose level and frequency, we expected that the sequences displayed very similar pharmacokinetics. Thus, the variable response in toxicity was more the result of the intrinsic toxicity of the sequence rather than differences in exposure.

**Figure 1.**
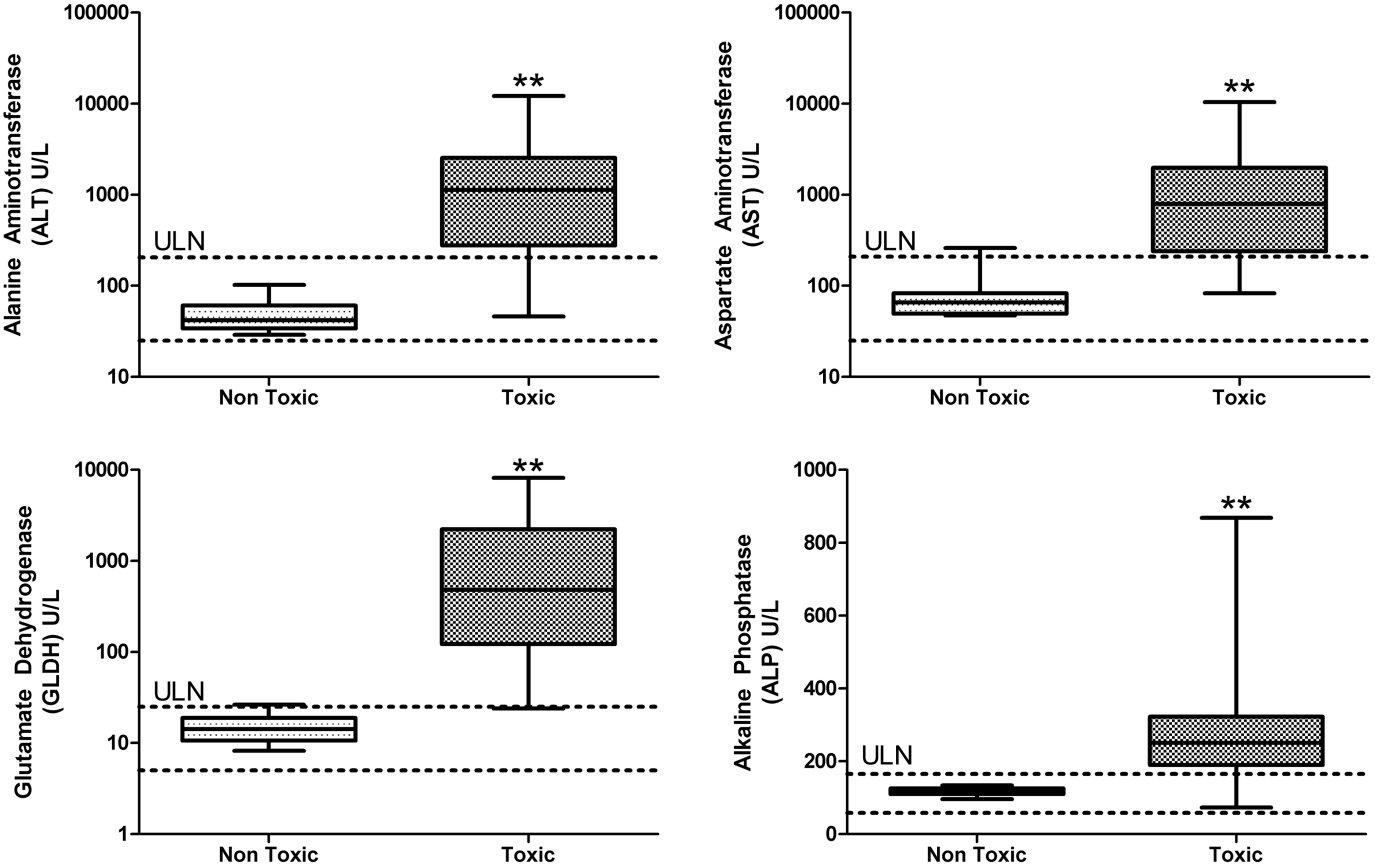
Comparison of liver enzyme values for 3-8-3 gapmers classified as non-toxic or toxic based on histopathology findings in the liver. Horizontal dashed lines represent the historical control values for each parameter in CD-1 mice (Pfizer internal data). Box plot demonstrates the median and 25th and 75th percentiles. Whiskers represent the maximum and minimum values in the dataset. ULN, upper limit of normal. **P < 0.01.

For the purposes of generating a classification model, we assigned sequences into categories of either toxic or non-toxic using histopathological changes in the liver. The histopathogy criteria used for this classification is further defined in the Materials and methods section*.* We compared this binary classification methodology using histopathology to standard biomarkers of hepatotoxicity such as serum ALT, AST, GLDH and ALP. As expected, the sequences classified as toxic by histopathology resulted in enzyme values generally much higher than the upper limit of normal (ULN) defined by internal historical data in CD-1 mice (Figure 1). Occasionally sequences were encountered that caused adverse microscopic changes in the liver but did not significantly elevate liver enzymes at day 15 (see full dataset in Supplementary Material). These sequences were still classified as toxic because with sufficient dose and/or duration of exposure we expected these would have caused more severe hepatotoxicity including increased liver enzyme values. For this reason, we believed that using histopathology scores was the most sensitive method to classify sequences as toxic or non-toxic. Consistent with previous reports, we ruled out target-mediated toxicity because many sequences were designed against the human mRNA and did not silence the corresponding target in the mouse (data not shown). Additionally, off-target antisense effects were considered unlikely since the sequences have very few predicted mouse target sequences with perfect homology.

**Figure 2.**
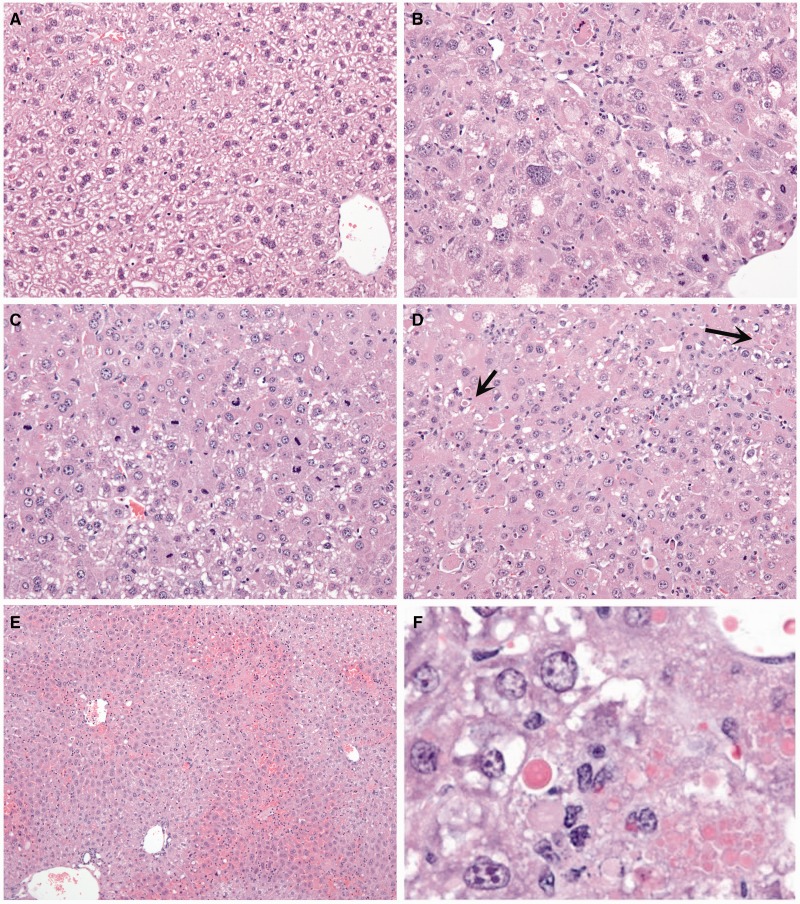
Liver histopathology observed after mice were treated for 2 weeks with 25 mg/kg 3-8-3 gapmers (two doses per week). (**A**) saline control (20X); (**B**) hepatocyte hypertrophy and karyomegaly (20X); (**C**) increased mitoses (20X); (**D**) single-cell necrosis/apoptosis (20X); (**E**) centrilobular hepatocellular necrosis (10X); (**F**) eosinophilic cytoplasmic alteration.

Morphological changes in the liver suggest there are significant alterations in transcriptional activity and protein production that precede cell death. The typical hepatic changes observed include apoptosis, single cell necrosis, increased mitoses, karyomegaly and hepatocellular hypertrophy. In rare instances, centrilobular to coalescing areas of necrosis are observed. Unlike 2′-MOE and earlier generation ASOs, hepatic inflammation is not commonly observed with LNAs, and if present, is most often secondary to necrosis. Examples of lesions observed included: hepatocellular hypertrophy and karyomegaly (Figure 2B), increased mitoses (Figure 2C), single-cell necrosis and apoptosis (Figure 2D), centrilobular hepatocellular necrosis (Figure 2E) and/or eosinophilic cytoplasmic alterations (Figure 2F). Any sequence that produced one or more of these lesions was classified as toxic for structure–toxicity assessments, independent of the magnitude of ALT/AST elevation.

### Identification of sequence motifs associated with hepatotoxicity

**Figure 3.**
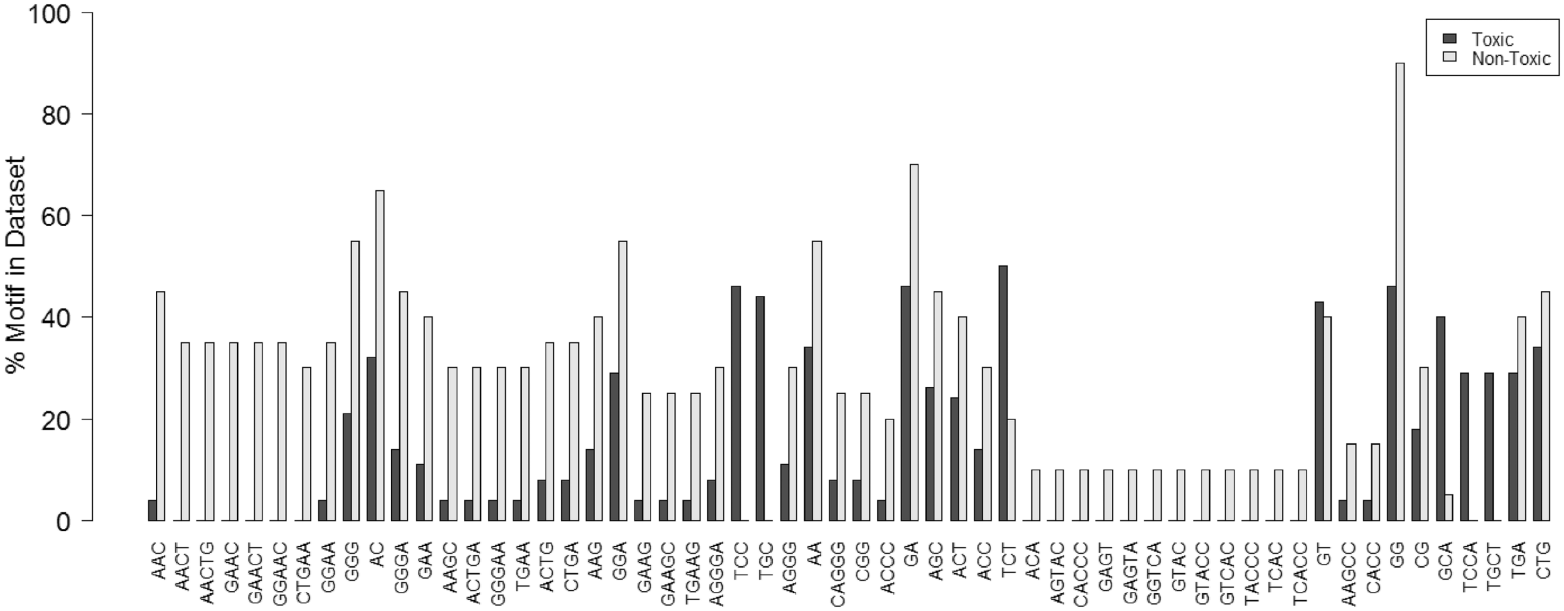
Distribution of toxic and non-toxic sequences in the selected 58 motifs: the TCC and TGC motifs show the strongest correlation with toxicity.

To test for structure–toxicity relationships, an algorithm to predict hepatotoxicity outcome based on nucleotide sequence was developed as described in Materials and methods section. First, a *χ*^2^ test using 1360 possible nucleotide permutations between two and five nucleotides in length identified 58 motifs that were statistically significant (*P* < 0.05), and those motifs were selected for further analysis (Figure 3). The refined list of sequence motifs was subsequently used to build a Random Forest (RF) classification model (20) to predict toxicity outcome of 71 3-8-3 LNA gapmers (51 toxic, 20 non-toxic). The model correctly classified 46/51 (sensitivity: 90%) toxic sequences and 11/20 (specificity: 55%) non-toxic sequences, based on the out-of-bag (OOB) estimated values. Interestingly, two sequence motifs were found to be overrepresented in toxic sequences. TCC and TGC motifs were among the most important variables in the RF model (second and 34th, respectively, using the ‘mean decrease accuracy’ output from the RF model). TCC, for example, was present in 18/51 toxic sequences while it never occurred in the non-toxic sequences. Likewise, for TGC, 17/51 toxic sequences had this motif and it similarly was never present in any non-toxic sequence. In other words, when the TGC and/or TCC were present in a sequence, the sequence was hepatotoxic in mice independent of the molecular target. Interestingly the presence of TGC and TCC motif and relationship to toxicity was independent of both position and LNA content. This is different from what was observed by Hagedorn *et al.* who observed a correlation between toxicity and LNA content in a motif (18).

To test the dependency of the TCC or TGC motifs for toxicity, several sequences were synthesized with minor variations to either introduce or remove the TGC and/or TCC motifs. These modified sequences were retested in mice as described previously. Introduction of TGC or TCC motifs into a non-toxic scrambled control sequence, **1a**, created the toxic sequences **1b**, **1d** and **1f** (Table 1). Unexpectedly, two sequences were created that did not cause liver lesions under the conditions tested in this study (**1c**, **1e**). Conversely, elimination of the TCC motif from the toxic *GR*-targeting sequence **4a** prevented toxicity in **4b** and **4d**, but not sequence **4c** (Table 1). The *Apoc3*-targeting sequence **3** contains both TGC and TCC and was one of the most highly toxic sequences tested in mice, causing morbidity and mortality ∼72 h after a single dose of 25 mg/kg. Overall, these data supported a strong relationship between the presence of TGC/TCC motifs and hepatotoxicity, but do not exclude the possibility that additional toxic motifs have not been identified based on our limited in-house dataset.

**Table 1.** Toxicity profile is altered by small changes in nucleotide sequence

ASO	Target	Sequence (5′–3′)	ALT (U/L)	AST (U/L)	Liver lesions
**1a**	None	TAATCGTCGATACC	28	38	No
**1b**	None	TAATGCTCGATCCC	3369.0	506	Yes
1c	None	TAATCCTCGATACC	31	43	No
1d	None	TAATCGTCCATACC	39	57	Yes
1e	None	TAATCGTGCATACC	39	55	No
1f	None	TAATCGTCGATGCC	48	51	Yes
**3**	ApoC3	TCAGTGCATCCTTG	–	–	Yes
4a	GR	AAGTCTGTTTCCCC	–	–	Yes
4b	GR	AAGTCTGTTTCACC	110	106	No
4c	GR	AAGTCTGTTACCCC	1943	1311	Yes
4d	GR	AAGTCTGTTACACC	49	74	No

A non-targeting control sequence 1a could be rendered toxic in 3/5 sequences where TGC and/or TCC motifs were introduced (1b–f). Conversely, a GR-targeting sequence 4a could be rendered non-toxic in 2/3 instances where the TCC motif was removed (4b–d).

### Motifs are associated with higher binding to hepatocellular proteins

**Figure 4.**
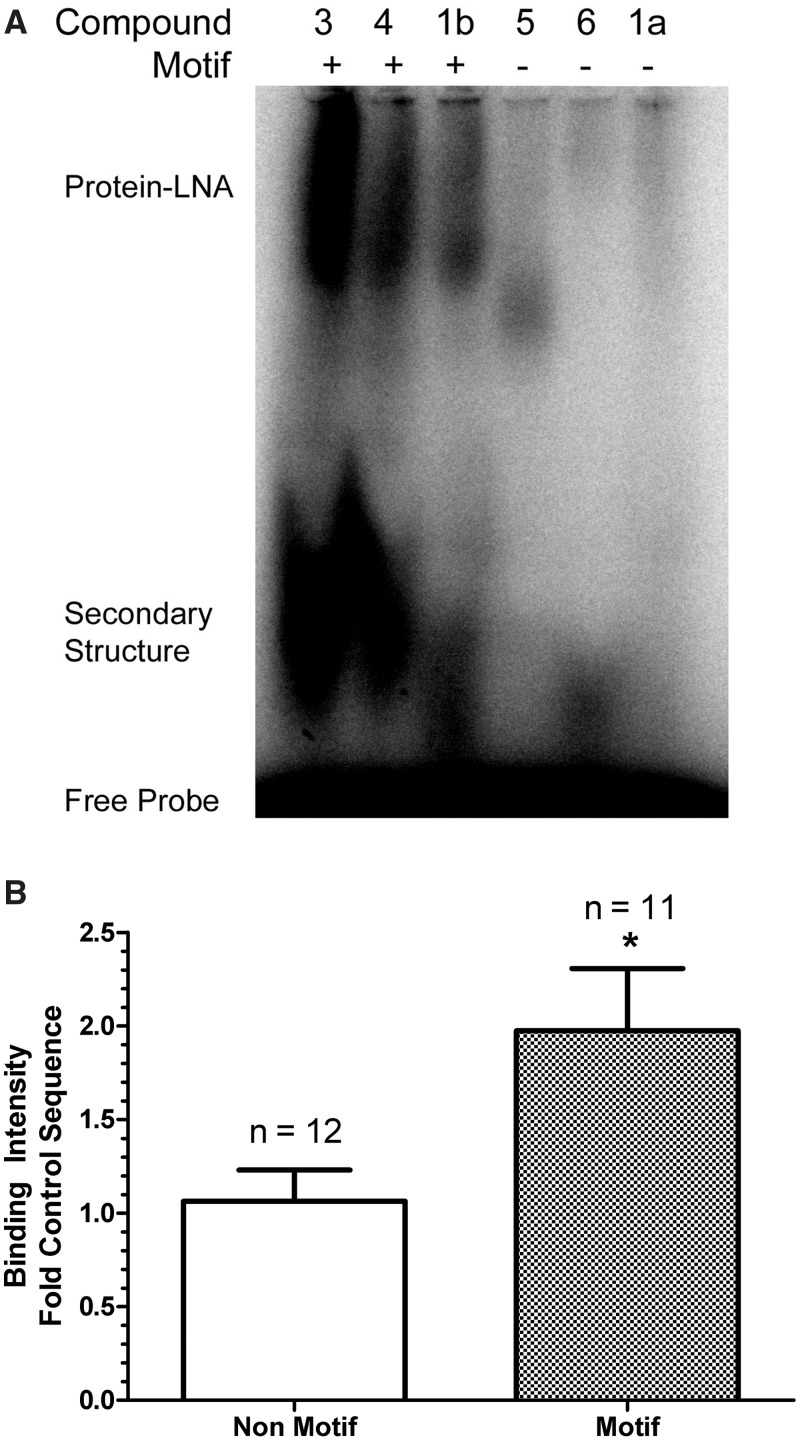
ASOs containing toxic sequence motifs TGC and/or TCC bind to hepatocellular proteins. (**A**) Representative gel of mouse liver protein homogenates were incubated with [γ 32P] ATP-labeled ASOs and protein complexes resolved by 6% native PAGE; (**B**) protein–LNA binding was determined for multiple non-toxic or toxic ASOs (*n* = 23) and binding intensities determined by phosphorimager analysis. Binding intensity is expressed as fold the non-toxic control sequence (1a) which was included on each gel to normalize binding intensity across experiments. *P < 0.05.

We hypothesized that toxic motifs could cause hepatotoxicity through mechanisms independent of antisense effects, such as disruption of cellular function by binding hepatocellular macromolecules. To this end, we evaluated if motif and non-motif sequences targeting *Apoc3* could form complexes with mouse liver proteins. When [γ ^32^P]ATP labeled 3-8-3 gapmers were and incubated with native mouse liver protein homogenates and resolved by 6% non-denaturing PAGE, high molecular weight protein–ASO complexes were detected (Figure 4A). Lower molecular weight bands were also detected in TGC and/or TCC containing ASOs, possibly representing additional secondary structure and/or ASO–ASO complexes. These structures may be important in mediating protein–ASO interactions as pre-heating ASO compounds prior to reaction setup eliminated these complexes and significantly reduced the appearance of protein–ASO complexes (data not shown). Significantly higher mean binding intensity (*P* = 0.02) was measured by image densitometry in motif-containing LNAs compared to non-motif containing sequences (Figure 4B). These data suggested that toxic TGC- and/or TCC-containing sequences have a higher propensity to complex with hepatocellular proteins compared to sequences without these motifs.

### Toxic motifs activate P53 and Nrf2 stress-responsive pathways in CD-1 mice

Because of the higher protein binding observed with motif-containing ASOs, we tested the potential for motif or non-motif containing sequences to differentially alter transcription *in vivo* using the FACTORIAL™, a multiplex reporter system enabling a simultaneous assessment of activities of multiple TFs within cells (24). The core component of the FACTORIAL™ system is a library of TF-inducible reporter constructs that, being transfected into evaluated cells, produce reporter RNAs proportionate to TFs activities. Thus profiling reporter RNAs’ concentrations within cells provides information about TFs activities (24). It was previously shown that this system can be efficaciously transfected into the mouse liver parenchyma cells *in vivo* by a hydrodynamic delivery (21). As the hydrodynamic transfection produces a transient liver injury, mice were allowed to recover for 2 weeks prior to dosing. During this recovery period, serum ALT and AST values return to baseline and the FACTORIAL™ reporters remain responsive to prototypical inducers (Attagene/Pfizer unpublished observations). After the recovery period, mice (10 males/group) received a single *s.c*. dose of LNA-modified ASOs (25 mg/kg) or a saline. Mice were euthanized 24 h after dosing and TF activities were measured as described in Materials and methods section.

**Figure 5.**
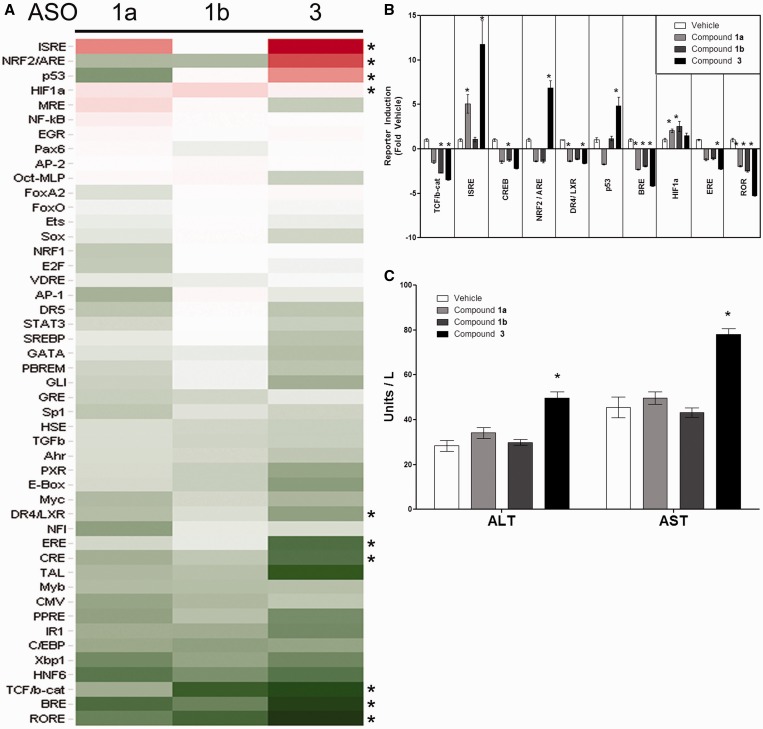
The impact of toxic and non-toxic 3-8-3 gapmers on TF activities in the mouse liver. (**A**) CD-1 male mice were transfected in vivo with FACTORIAL™ reporter plasmid library using a hydrodynamic transfection thru the tail vein. Two weeks later, mice were divided into groups (*N* = 10 animals/group) and treated with a single 25 mg/kg s.c*.* dose of 1a, 1b or 3 or with saline. Twenty-four hours later, livers were collected and reporter RNAs and corresponding TF activities in the livers were assessed as described (23,21). (**B**) Statistically significant TF activity changes (ANOVA, *P* < 0.05) in ASOs-treated relative to vehicle-treated mice are shown. (**C**) Serum ALT and AST in mice 24 h after dosing with 25 mg/kg 1a, 1b or 3. Bars represent the mean ± SEM, *P < 0.05.

The compounds selected for this study included two hepatotoxic sequences containing the TGC and TCC motifs (Compound **3** and **1b**) and a non-toxic, non-targeting control sequence without a motif (**1a**) (Table 1). While **3** and **1b** are hepatotoxic in mice, they differed in severity; **3** was more toxic and resulted in morbidity and mortality ∼72 h after a single dose at 25 mg/kg, whereas **1b**-treated mice survived for the duration of the study (two doses per week for 2 weeks). In this article, the time point of 24 h was chosen to capture TF responses that occurred prior to overt hepatotoxicity, as well as to allow mice treated with compound **3** to be euthanized before physical signs of toxicity became apparent. Of the 46 analyzed TFs, 10 TFs were significantly (*P* < 0.05) activated or inhibited relative to saline treated mice (Figure 5). The P53 and Nrf2/ARE reporters were substantially induced by compound **3** but not by **1a** or **1b**, suggesting these pathways may be associated with motif-related hepatotoxicity. Although **1b** contains the TGC and TCC motifs and was hepatotoxic after repeated dosing, it did not elevate AST/ALT after a single dose at 25 mg/kg (Figure 5C), consistent with previous *in vivo* data. Therefore, the absence of Nrf2 and P53 induction by **1b** was not unexpected at this early time point.

## DISCUSSION

ASOs with LNA modifications offer advantages for improved target specificity and potency, but are hampered by an increased likelihood of hepatotoxicity in non-clinical studies. In pursuit of LNA-modified ASO therapeutics targeting human *Apoc3*, *Crtc2* or *GR* several promising sequences were evaluated in mice in order to identify one or more safe clinical candidates. Because a high percentage of these sequences were found to be hepatotoxic, we investigated if structure–toxicity approaches could be used to predict toxicity based on nucleotide sequences, and reduce the need for extensive animal studies.

Sequence motifs within a set of ∼71 ASO 3-8-3 gapmers were tested for associations with toxicity in mice. The 14-mer sequences were deconvoluted into sequence motifs from 2 to 5 nucleotides in length. Of the 1360 possible permutations of A, G, C and T, 58 were found to have a significant association with hepatotoxicity (*χ*^2^ test, *P* < 0.05). These statistically significant motifs were then used to generate a classification model that could be utilized to predict toxicity outcome based on motifs. The RF predictor generated from these data correctly classified 90% of the toxic sequences and 55% of the non-toxic sequences. Two motifs (TGC and TCC) were found to be strongly associated with hepatotoxicity in mice. Of the 51 toxic sequences in the test set, 27 of these sequences contained TGC and/or TCC motifs. The remaining toxic sequences which did not contain these sequences may contain other important motifs that were not well represented in the training set and classification model. Unexpectedly, two sequences were created during the model validation stage that did not cause liver lesions despite having TCC or TGC motifs (Table 1, **1c**, **1e**). These represented the only two motif-containing compounds that were not classified as toxic under our testing paradigm. These sequences may have represented less toxic compounds that would have been classified as toxic in longer term studies or studies conducted at a higher dose. Also the sequences that were utilized in identification of the TCC and TGC motifs all underwent *in vitro* screening for potency that might have directed them to a subcellular compartment which could be different in mutated sequences that did not show toxicity with presence of these motifs as these altered sequences did not go through a similar screening. Alternatively, the location of these motifs in the sequence may be protected or insufficient for the initiation of hepatotoxicity.

To explain the toxicity of TGC/TCC motifs, we hypothesized that these sequences could bind to hepatocellular macromolecules, possibly leading to altered function and cellular death. To detect if LNA-modified ASOs would associate with hepatocellular proteins, we incubated mouse liver homogenates with 5′-[γ ^32^P]ATP-labeled ASOs and then resolved the proteins with native PAGE (Figure 4A). Indeed, increased binding of ASOs containing TGC and/or TCC was observed compared to ASOs without these motifs (Figure 4B). Interestingly, one of the most highly toxic sequences in mice, compound **3**, contained both motifs and was also the strongest protein binding sequence. In addition to higher molecular weight bands attributed to protein–ASOs, we detected lower molecular weight complexes migrating near the unbound ASOs. We have speculated that these are either mutimeric complexes or single stranded ASOs with secondary structure (e.g. hairpins), since they can be completely disrupted by heat denaturing the labeled ASOs prior to electrophoresis. These structures may be important in mediating the protein–ASO interactions because eliminating these complexes prior to incubation with mouse liver proteins significantly reduces the protein–ASO-binding intensity. The relationship of these complexes to toxicity *in vivo* was not directly tested because we did not investigate specialized formulations or methods necessary to prevent these complexes from forming in the dosing solution. It is possible that simple procedures, such as heat denaturing the dosing solution proior to administration, could be sufficient. However, prior to initiating these experiments methods should be in place to ensure that the heat denaturation of the oligonucleotides results in a sustained loss of the species both in the dosing formulation as well as *in vivo* after test article administration. Without being able to definitively demonstrate this it would be difficult to interpret a result in which the oligo was still toxic. The formation of ASO–protein complexes does occur in some non-toxic sequences, and we suspect that the complexes identified thus far are heterogeneous. Experiments to identify the ASO–protein complexes are currently underway to discriminate toxic from non-toxic-binding partners, but are complicated by the methodology required to label the ASO and capture sufficient quantity of proteins.

To begin to elucidate the molecular events leading up to histopathological changes and overt liver toxicity in mice, we tested for changes in the basal activities of TFs in the mouse livers after being treated with ASOs. To accomplish this, we used the FACTORIAL™ system, a multiplex reporter assay developed by Attagene Inc. (24). Livers of mice were transduced with a library of reporter plasmids by hydrodynamic transfection thru the tail vein (22,23). Following recovery, mice were randomized into four groups and administered saline or a single s.c. dose of 25 mg/kg **1a**, **1b** or **3** for a short duration (24 h).

Of the 46 TF-responsive reporters of the FACTORIAL™ library, four reporters (ISRE, Nrf2/ARE, P53 and HIF1) were significantly upregulated by LNA gapmers when compared to saline treated controls (Figure 5). However, only Nrf2/ARE and P53 reporters were differentially induced by **3**. The activation of the P53 pathway by hepatotoxic LNA gapmers is intriguing since P53 transcriptional activity is known to be enhanced by the double-stranded RNA (dsRNA)-dependent protein kinase PKR (25). PKR recognizes viral RNA via two RNA-binding motifs (RBMs) in the N-terminal region resulting in phosphorylation and dimerization to an active enzyme (reviewed in (26)). When activated, PKR in turn phosphorylates eukaryotic translation initiation factor 2 alpha (EIF2α) at serine 51 inhibiting its function. A role for PKR for mediating hepatotoxicity of LNA-modified ASOs has been suggested previously, since enhanced phosphorylation of EIF2α was observed with hepatotoxic LNA ASOs (17). Histological evidence indicating that hepatotoxicy by LNAs is due to hepatocellular apoptosis and single-cell necrosis, without a primary inflammatory response, is consistent with the premise that liver injury occurs through initiation of apoptosis in the hepatocyte. Here, we provide further evidence to suggest that toxicity of LNAs might be mediated through initiate antiviral responses that might be mediated through P53 pathways and are somehow specific to LNA-modifications.

Although **1b** was also hepatotoxic in mice, the onset of toxicity was later and less severe than **3**. This difference in the toxicity severity between the two compounds likely accounts for the differences in P53 activity at the 24-h time point. Liver samples from mice treated for 2 weeks with **1b** were analyzed by whole-genome microarrays and we detected gene signatures consistent with enhanced P53 activity (e.g. upregulated *cyclin D1*, and *cyclin-dependent kinase inhibitor 1A*, unpublished observations). However, when liver samples were analyzed after only 24 h of treatment with this oligonucleotide, very few differentially regulated genes were detected. Pathway analysis of mice treated for 24 h with **3** was remarkably similar to that of **1b** after 2 weeks, suggesting that these compounds share similar mechanisms leading to up to hepatocellular injury (unpublished observations). Of note, both **1a** and **3** induced the interferon response element in the FACTORIAL™ assay. Compound **1a** contains a methylated CpG to reduce Toll-like receptor activity; however it is plausible that some level of interferon induction remains with this modification. Interferon responses may be important in mediating hepatotoxic responses to LNAs, however, because **1a** was not hepatotoxic in mice treated beyond 2 weeks it is not likely the sole driver of toxicity. Additional experiments to explore differences in the interferon responses to **1a** and **3** would be necessary to determine if these responses are obligate for toxic responses to LNA-modified ASOs.

In conclusion, we have demonstrated that hepatotoxicity of LNA-modified ASOs is strongly associated with TGC and TCC sequence motifs in 3-8-3 gapmers. ASOs containing these sequence motifs tend to exhibit higher binding to mouse liver proteins. These ASO–protein complexes could inactivate critical cellular processes and/or may activate hepatocellular antiviral responses resulting in apoptosis. One such pathway could be the P53 pathway, which was activated 24 h after a single dose of 25 mg/kg compound **3**. Consistent with a previous report (18) we have demonstrated the utility of using structure activity modeling in order to predict hepatoxicity of oligonucleotides. In addition, we provide new evidence that the presence of certain structural motifs is associated with biochemical outcomes, such as protein binding and transcriptional activation. These results further suggest it is possible to eliminate hepatotoxic LNA-modified ASOs at the design stage, improve *in vitro* screening models, and to limit the number of animals needed for tolerability screening.

## SUPPLEMENTARY DATA

Supplementary Data are available at NAR Online.

## FUNDING

Funding for open access charges: Pfizer Inc.

*Conflict of interest statement*. None declared.

## Supplementary Material

Supplementary Data
