# Supplementary material for: Sequence motifs associated with hepatotoxicity of locked nucleic acid—modified antisense oligonucleotides
Source: Nucleic Acids Res. 2014 Feb 18;42(8):4882–91. doi: 10.1093/nar/gku142 (PMC4005641; doi:10.1093/nar/gku142)
Supplement: Supplementary Data [file supp_42_8_4882__index.html]

Sequence motifs associated with hepatotoxicity of locked nucleic acid—modified antisense oligonucleotides — Sequence motifs associated with hepatotoxicity of locked nucleic acid—modified antisense oligonucleotides — Supplementary Data 

# Sequence motifs associated with hepatotoxicity of locked nucleic acid—modified antisense oligonucleotides

## Supplementary Data

files

**Files in this Data Supplement:**

- Supplementary Data - xls file
